# Do Community Free-Medication Service Policy Improve Patient Medication Adherence? A Cross-Sectional Study of Patients With Severe Mental Disorders in Beijing Community

**DOI:** 10.3389/fpubh.2021.714374

**Published:** 2021-07-26

**Authors:** Junli Zhu, Qingzhi Huang, Wei Lu, Yun Chen, Bin Li, Ying Xu, Rui Xi, Dan Li

**Affiliations:** ^1^School of Public Health, Capital Medical University, Beijing, China; ^2^Research Center for Capital Health Management and Policy, Beijing, China; ^3^The National Clinical Research Center for Mental Disorders & Beijing Key Laboratory of Mental Disorders, Beijing Anding Hospital, Capital Medical University, Beijing, China; ^4^Advanced Innovation Center for Human Brain Protection, Capital Medical University, Beijing, China; ^5^Beijing Institute of Mental Health, Beijing, China

**Keywords:** severe mental disorder, medication adherence, community free-medication service policy, community management, intervention

## Abstract

**Background:** Nowadays, mental health problems have become a major concern affecting economic and social development, with severe mental health disorders being the top priority. In 2013, Beijing began to implement the Community Free-Medication Service policy (CFMS). This article aims to evaluate the effect of the policy on medication adherence.

**Methods:** In this study, multi-stage sampling was used to select representative patients as samples. Some of the baseline data were obtained by consulting the archives, and information about patient medication adherence measured by Brooks Medication Adherence Scale was obtained through face-to-face interviews. Logistic regression was used to examine the impact of the policy.

**Results:** Policy participation had a significant positive impact on medication adherence (OR = 1.557). The effect of policy participation on medication adherence in the Medication-only mode and Subsidy-only mode were highly significant, but it was not significant in the Mixed mode.

**Conclusion:** This study found that the CFMS in Beijing as an intervention is effective in improving the medication adherence of community patients. However, the impact of the policy is not consistent among service modes. Reinforcement magnitude and frequency should be considered when designing reinforcement interventions.

## Introduction

Mental health problems have become major public health and social problems, affecting economic, and social development. A study by the Harvard School of Public Health projected that between 2011 and 2030, mental disorders will cause up to 16.3 trillion US dollars in economic output losses worldwide (1). Mental health disorders are often related to other diseases such as cancer, cardiovascular disease, diabetes, stroke, tuberculosis, and HIV/AIDS. Poor adherence to antipsychotic drugs is a major problem in the treatment of psychotic disorders. Previous many literature have shown between 25 and 80% of patients fail to take their drugs correctly at some point in their treatment (2) and poor adherence has been strongly associated with a higher risk of relapse, increased hospitalisation rates, lower rates of remission of positive symptoms and a poorer quality of life (3, 4). And Knapp et al. also found that poor adherence to treatment can treble the costs of external services treble the costs of external services (5). In 2018, among the six types of patients with severe prudential disorder registered in China, patients with two-way affection had the highest regular medication rate, only 48.98% (6).

In 2010, the World Health Organisation called mental health patients a vulnerable group, and in the “2013–2020 Comprehensive Mental Health Action Plan” adopted by the Sixty-sixth World Health Assembly, it was clearly stated that the government is the ultimate guardian of the population's mental health and should assume the main responsibility. Data from the Chinese CDC have shown that mental disorders rank highest in the burden of disease in China, accounting for about 20% of the total disease burden (7). Severe mental disorders include six major mental disorders including schizophrenia, schizoaffective disorder, paranoid psychosis, bipolar disorder, mental disorders caused by epilepsy, and Intellectual Disability with mental disorders (8). At the end of 2018, there were 6 million patients registered with severe mental disorders in China, and the reported prevalence rate was 0.43% (6). Huang et al. estimated that mental illness will reduce China's productivity by more than $900 million between 2012 and 2030 (9). The report of the 19th National Congress of the Communist Party of China put forward the “Healthy China Strategy,” which states that the Chinese government will implement mental health promotion actions, and the severe mental disorders will be effectively prevented and controlled by 2022.

In September 2004, the General Office of the State Council of China issued the “Notice on Further Strengthening the Guidance of Mental Health Work,” which clarified that a multi-channel fund-raising mental health model with government investment as the mainstay will be established to carry out treatment and provide assistance for patients with mental illness, promoting the prevention and control of mental illness. In December 2004, the national “686 Program,” named after the initial funding allocation of 6.86 million Yuan (Chinese currency; equivalent to US$1 million today) and also called “Central Government Support for the Local Management and Treatment of Serious Mental Illness Project,” was initiated to integrate hospital and community services (10, 11). A systematic review of 57 trials treating a variety of psychiatric illnesses has found that integrated chronic care models can improve mental and physical outcomes for individuals with mental disorders across a wide variety of care settings without increasing cost (12). At the beginning of 2009, Chaoyang District took the lead in issuing the “Subsidy Program” in Beijing and provided subsidies to poor patients with Chaoyang household registration (13). And then, Pinggu District also carried out the “Free Treatment Program” in July 2012. But it extends the service population to all patients with mental disorders for all mental disorders. In October 2013, the Beijing Municipal Health Bureau issued the “Administrative Measures for the Treatment of Severe Mental Disorders Using Free Essential Medicines in Outpatient Clinics (Trial)” (14). The Community Free-Medication Service policy (CFMS) proposes that the district government shall provide free basic medicine treatment services to patients with all 6 types of severe mental disorders with Beijing household registration. The procedures for participating in the policy are as follows: Firstly, Those who have been diagnosed with severe mental disorders by psychiatric hospitals submit relevant materials including diagnosis and treatment, household, and others to the community medical institution, which will be reviewed by the district government's psychiatric prevention department. Secondly, the psychiatrist of the community institution conducts baseline information statistics on the patients who have passed the audit, and establishes a personal file. Thirdly, the patients or their families regularly visit designated medical institutions to receive free medicine, and receive the guidance of primary care psychiatrists. At the end of 2014, the service was fully launched in 16 districts in Beijing. In the specific implementation process, Beijing's 16 districts actively explored and formed a policy mode that was suitable for the district. In terms of service methods, Haidian, Chaoyang, and Tongzhou districts not only provide free medicines to patients, but also provide appropriate subsidies or reimbursements for medicines with receipts. Fengtai District only provides financial subsidies, 200 Yuan per person per month, and the patient chooses the location where they buy the medicine. The remaining twelve districts implement the mode of simply receiving medicine. Later, there were also seven cities or districts in other provinces implemented CFMS for severely mental patients.

One of the most important aims of China's CFMS is to improve the adherence of patients with severe mental disorders, improve patient reluctance to take medications due to economic burden, and improve the social function of patients (15). The previous literature has shown that demographic factors (16), as well as other factors including family support and cognition of the disease (17), are also influencing factors. In the specialist hospital, a variety of interventions have a positive effect on promoting rehabilitation, and the government department in charge of mental health should further integrate these interventions to benefit more patients (18, 19). It is also indispensable for the government to intervene in medication adherence by means of health policies. For example, Chile introduced a policy in 2006 to provide financial protection for four types of mental illness (20).

There are only a few publications researching the policy effects of CFMS in China, two of which are in English. Li et al. analysed the effects of the 686 project with 3,090 participating patients in Mianyang, Sichuan as the research object (21). Although the research object is different from Li et al. (21), Gong et al. also studied the effect of the 686 project (15). The remaining papers are in Chinese. In most areas with CFMS, the service population only includes poor patients with severe mental disorders in their jurisdictions, and a few areas have expanded to all patients with severe mental disorders in their region. The remaining literature are primarily based on poor mental patients and the sample size is <200. After the launch of the 686 project, Beijing, as the capital of China, took the lead in expanding CFMS to all household-registered patients with severe mental disorders. As the cumulative population of patients has increased in recent years, according to the Beijing Municipal Health Commission, in 2020, the district government's financial investment for this service reached 89.85 million Yuan, which is 8.22 times the initial amount invested in 2013. By receiving free basic medicines from primary medical institutions every month, the opportunity for patients and their families to meet with doctors for prevention and treatment was increased. During the interview, patients are guided and urged to take medication regularly, meaning that the importance of long-term medication is strengthened (22). The previous literature has introduced or evaluated the community management of patients with mental disorders in developed countries, such as FSP (Full Service Partnership) programs in the United States (23), ACT (Assertive Community Treatment) programs in the United Kingdom (24), and CTO (Community Treatment Orders) in Canada (25), but few in developing countries. Beijing, as the capital of China, its CFMS policy maybe provide a policy intervention experience in developing countries. However, there has not yet been a study published on the impact of Beijing's CFMS on patient adherence to medication. It is necessary to conduct systematic investigations and studies to evaluate the impact of policy implementation on medication adherence, and to provide a reference for policy adjustments.

## Materials and Methods

### Sample and Data

There were two methods used to collect data in this study. The baseline data, including demographics, disease treatments, and family of the patients, were collected from the Beijing Municipal Management Information System for Patients with Severe Mental Disorders. In contrast, data on antipsychotic medication adherence were collected from the registered patients through a questionnaire, which was primarily in the form of face-to-face interviews and with telephone interviews for respondents who have difficulty in moving. All respondents in the study were voluntary and written informed consent was obtained. To maintain confidentiality, names were not required on the questionnaires. The investigators were undergraduate or graduate students of clinical and public health majors of Capital Medical University who had been trained to master questioning skills and communication methods with patients with mental disorders and their families. In addition, a unified simulation training scenario was carried out before the survey. This study passed the ethical review conducted by the Medical Ethics Committee of Capital Medical University.

**Figure 1:**
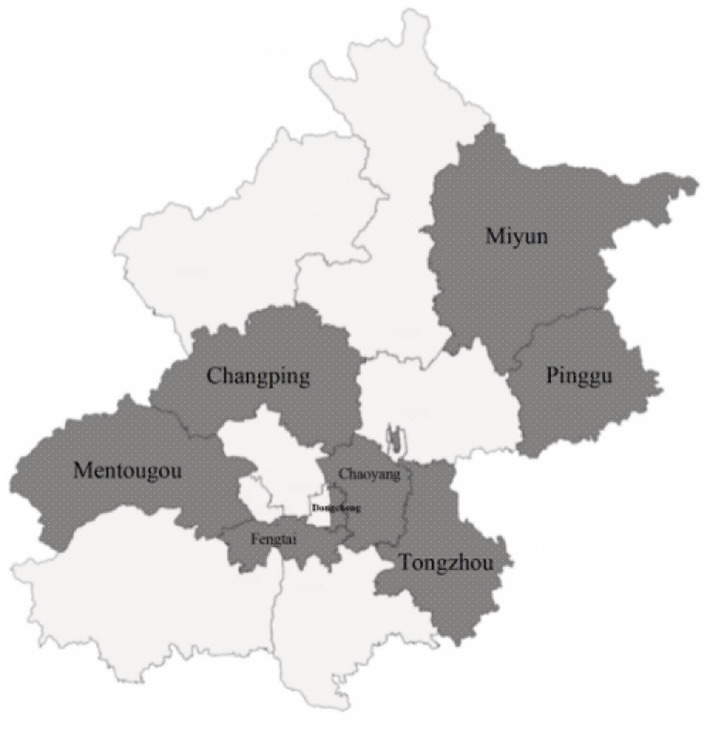
Districts sampling situation.

The samples were obtained by multi-stage stratified sampling using the following steps: first, there are three modes of providing CFMS in the 16 districts of Beijing—“Subsidy-only mode,” “Mixed mode,” and “Medication-only mode.” We divided the 16 districts into three groups according to the mode applied. Second, the sample districts were selected. Fengtai District was selected as the sample because it was the only district that adopted a pure subsidy model. In the mixed service model group, Tongzhou District was selected as the sub-centre of Beijing. Then, we randomly took one sample district from the remaining Chaoyang District and Haidian District, which are similar in economic development level and topographical characteristics. For the twelve districts of the “Medication-only mode” group, we divided them into three sub-groups according to the level of economic development and then randomly took one sample district from those with a per capita GDP of more than 150,000 Yuan (DongCheng, XiCheng, and ShunYi), three from the districts with a per capita GDP of 50,000 Yuan to 150,000 Yuan (ShijingShan, HuaiRou, FangShang, MiYun, MentouGou, and PingGu), and one from the districts with a per capita GDP of 50,000 Yuan (YanQing, Changping, and DaXing). The selected districts is shown in Figure 1. Third, each district taken above was stratified based on topographical features. Mentougou District was not stratified because of its entirely mountainous area, and each of the other districts was divided into plain and mountainous areas according to the topographical features. Fourth, the sample communities were taken; only one community was sampled in Mentougou District, and one was sampled in the plains and mountainous areas of other districts, meaning that 15 communities were finally sampled. Finally, cluster sampling was performed on all patients on file who met the inclusion criteria in the selected communities.

The inclusion criteria for survey subjects of this study are as follows: (1) patients meet the ICD-10 standard and have been diagnosed with one of the six types of severe mental disorders; (2) health records have been established in the community; and (3) currently not hospitalised and managed normally in the community. The following exclusion criteria were applied: (1) the patient is unable to complete the survey due to insufficient cognitive ability or communication difficulties, and family members cannot assist in completing the survey due to their age or not being local; (2) the patient has a high risk of suicide; and (3) refusal to accept their disease or currently refusing to accept community management due to other reasons. The survey lasted 3 months from August to October 2020. There were 2,240 patients on file in the 15 communities, of which 1,710 responded, accounting for 76%. At the same, the response rate of each community in each district was no <75%. After deleting respondents without medication based on the doctor's prescription and with missing values, the final sample consisted of 1,641 patients.

### Instruments and Models

The study's dependent variable is medication adherence, but there is no gold standard for judging it (26). There are hundreds of scales for measuring medication adherence in the previous literature (27) but considering that the energy of this type of special population is not enough to answer too many items, we use the 4-item Brooks Medication Adherence Scale (28) which have shown high levels of reliability (Cronbach's α = 0.78) and construct validity, meanwhile the measurement period is the past 12 months. This scale was used by Tamburrino et al. in the study of medication adherence in patients with depressive disorder (29). According to the method of Tamburrino et al. and Defaru et al. patients who choose “No” for all items are defined as having good medication adherence, while patients who choose “Yes” for one or more items are considered to have poor medication adherence (29, 30). Several steps were used to select items for the questionnaire. First, we translated the items from the existing English instruments into Chinese and replaced with “severe mental disorder,” “antipsychotic drugs” according to the research theme. Second, expert consultation including four professors from Mental Health and Health Economics, three clinical doctors from psychiatric hospitals, and three psychiatrists from primary medical institutions, who will provide assistance in scrutinising the appropriateness. Then, a pre-survey was executed with a small sample to modify the instruments.

The purpose of this study was to evaluate the effect of the policy, meaning that the “whether to participate policy” is the most important independent variable. The “policy participation” is defined as: from January 1 to December 31, 2019, patients have completed the policy procedures and can receive medicine or subsidies on a regular basis. Based on the previous literature, we also selected other independent variables, including 15 variables divided into 5 categories: patient sociological characteristics, disease-related features, self-feeling factors, family support factors, and social support factors. Przemyslaw et al. and Nie et al. pointed out that some personal characteristics such as being married, employment and a higher education level are factors that promote medication adherence (16, 31), while Wu et al. found that different disease-related features such as diseases and courses had significant differences in medication adherence (32). Ram and Gowdappa research showed that self-feeling factors such as symptoms and side effects also have an impact on medication adherence (33). Hernandez et al. showed that family support has an important position in medication adherence, while Nose et al. demonstrated that social support is also significant (17, 34]. In the end, based on extensive reading of relevant literature, combined with the actual situation in China, these 15 representative variables were determined and various parameters were set.

Descriptive statistics of the study variables are reported using frequencies and percentages. To examine the impact of CFMS and other factors on the measures of medication adherence, there are two steps in statistical analysis. The first step is to use chi-square test to perform a univariate analysis of the basic characteristics and medication adherence. The second step is multivariate logistic regression. The model is divided into two categories: one is for the overall sample, and the other is grouped for regression analysis. As described above, there are three modes of the policy in Beijing districts. In order to test whether the policy effects of each model are the same, in addition to the multiple logistic regression for the full sample, logistic regression is also performed on the samples of each mode. All statistical analysis is achieved through SPSS 26.0.

## Results

### Description of the Basic Characteristics of the Sample

The basic characteristics of the overall respondents in this study are shown in Table 1. 948 (57.8%) had good medication adherence within 12 months. The file records shows that 1,348 respondents (82.1%) participated in the CFMS. The majority of respondents were female (52.6%), and married (56.8%). The proportions of respondents suffering from schizophrenia (SCH), bipolar disorder (BD), and Intellectual Disability with mental disorders (ID-MD) are 57.0, 15.1, and 17.9%, respectively, with 79.8% having a disease course of 10 years or longer. Policy implementation mode districts are divided into three groups of patients: 39.3% from the Medication-only mode, 19.3% from the Subsidy-only mode, and 41.4% from the mixed mode. The characteristics of the respondents of the three groups and policy participation situation are also shown in Table 1.

**Table 1:** Description of respondents characteristics.

**Variable**		**Overall (*n* = 1,641)**	**Policy participation**		**Policy Mode**		
			**Non-participate (*n* = 293)**	**Participate (*n* = 1,348)**	**Medication-only (*n* = 645)**	**Subsidy-only (*n* = 316)**	**Mixed (*n* = 680)**
**Medication adherence**	Good	948 (57.8)	141 (48.1)	807 (59.9)	379 (58.8)	200 (63.3)	369 (54.3)
**Personal characteristics**							
Sex	Female	863 (52.6)	159 (54.3)	704 (52.2)	359 (55.7)	148 (46.8)	356 (52.4)
Marital	Married	932 (56.8)	146 (49.8)	786 (58.3)	377 (58.4)	188 (59.5)	367 (54.0)
Education	Primary	530 (32.3)	85 (29.0)	445 (33.0)	184 (28.5)	102 (32.3)	244 (35.9)
	Junior	672 (41.0)	112 (38.2)	560 (41.5)	268 (41.6)	127 (40.2)	277 (40.7)
	High	439 (26.8)	96 (32.8)	343 (25.4)	193 (29.9)	87 (27.5)	159 (23.4)
Professional	Employed	197 (12.0)	39 (13.3)	158 (11.7)	74 (11.5)	28 (8.9)	95 (14.0)
**Disease-related features**							
Diseases	SCH	936 (57.0)	176 (60.1)	760 (56.4)	434 (67.3)	164 (51.9)	338 (49.7)
	BD	248 (15.1)	48 (16.4)	200 (14.8)	88 (13.6)	56 (17.7)	104 (15.3)
	ID-MD	293 (17.9)	37 (12.6)	256 (19.0)	60 (9.3)	71 (22.5)	162 (23.8)
	Others	164 (10.0)	32 (10.9)	132 (9.8)	63 (9.8)	25 (7.9)	76 (11.2)
Physical condition	Sick	254 (15.5)	28 (9.6)	226 (16.8)	105 (16.3)	69 (21.8)	80 (11.8)
Course of disease	≤ 9	332 (20.2)	63 (21.5)	269 (20.0)	90 (14.0)	88 (27.8)	154 (22.6)
	10–29	817 (49.8)	135 (46.1)	682 (50.6)	337 (52.2)	167 (50.9)	319 (46.9)
	≥30	492 (30.0)	95 (32.4)	397 (29.5)	218 (33.8)	67 (21.2)	207 (30.4)
Types of medicine	≤ 1 type	790 (48.1)	196 (66.9)	594 (44.1)	224 (34.7)	192 (60.8)	374 (55.0)
	2 types	475 (28.9)	58 (19.8)	417 (30.9)	205 (31.8)	86 (27.2)	184 (27.1)
	3 types	255 (15.5)	29 (9.9)	226 (16.8)	134 (20.8)	35 (11.1)	86 (12.6)
	≥4 types	121 (7.4)	10 (3.4)	111 (8.2)	82 (12.7)	3 (0.9)	36 (5.3)
**Self-feeling factors**							
Symptoms	Unstable	508 (31.0)	106 (36.2)	402 (29.8)	177 (27.4)	103 (32.6)	228 (33.5)
Side effects	Frequently	272 (16.6)	54 (18.4)	218 (16.2)	107 (16.6)	71 (22.5)	94 (13.8)
**Family support factors**							
Financial situation	Non-poverty	1,046 (63.7)	164 (56.0)	882 (65.4)	329 (51.0)	271 (85.8)	446 (65.6)
Co-resident	Yes	1471 (89.6)	244 (83.3)	1227 (91.0)	622 (96.4)	304 (96.2)	545 (80.1)
**Social support factors**							
Medication guidance	Getting	417 (25.4)	47 (16.0)	370 (27.4)	188 (29.1)	49 (15.5)	180 (26.5)
Medicine location	Community	570 (34.7)	15 (5.1)	555 (41.2)	200 (31.0)	24 (7.6)	346 (50.9)
Years of participation in community archives	≤ 5	548 (33.4)	103 (35.2)	445 (33.0)	168 (26.0)	155 (49.1)	225 (33.1)
	6–10	738 (45.0)	134 (45.7)	604 (44.8)	368 (57.1)	130 (41.1)	240 (35.3)
	≥11	355 (21.6)	56 (19.1)	299 (22.2)	109 (16.9)	31 (9.8)	215 (31.6)

### Variables Assignments and Univariate Analysis Results

The variable assignment situation and the univariate analysis affecting medication adherence are shown in Table 2. Compared with non-participants, patients participating in the policy have better medication adherence (*p* < 0.01), and statistical significance was among different policy modes (*p* < 0.05). Among other characteristics, patients with different education levels, types of diseases, physical condition, types of medicine, self-perception of symptoms and side effects, and medicine location are statistically significant in medication adherence.

**Table 2:** Variables assignments and univariate analysis results.

**Variable**		**Assignment**	**Medication adherence**		**Good medication adherence rate(%)**	**Chi-square**
			**Poor(*n* = 693)**	**Good(*n* = 948)**		
**Policy participation**						
Non-participate		0	152	141	48.1	13.606^***^
Participate		1	541	807	59.9	
**Policy Mode**						
Medication-only		/	266	379	58.8	7.632^**^
Subsidy-only		/	116	200	63.3	
Mixed		/	311	369	54.3	
**Personal characteristics**						
Sex	Female	0	372	491	56.9	0.571
	Male	1	321	457	58.7	
Marital	Single	0	302	407	57.4	0.068
	Married	1	391	541	58.0	
Education	Primary	Assign a value of 1–3 from low to high	245	285	53.8	4.479^**^
	Junior		275	397	59.1	
	High		173	266	60.6	
Professional	Completely unemployed	0	610	834	57.8	0.001
	Employed	1	83	114	57.9	
**Disease-related features**						
Diseases	SCH	Set 3 dummy variables	381	555	59.3	27.320^***^
	BD		88	160	64.5	
	ID-MD		162	131	44.7	
	Others		62	102	62.2	
Physical condition	Normal	0	604	783	56.5	6.370^**^
	Sick	1	89	165	65.0	
Course of disease	≤ 9	Assign a value of 1–3 from short to long	146	186	56.0	0.100
	10–29		329	488	59.7	
	≥30		218	274	55.7	
Types of medicine	≤ 1 type	Assign a value of 1–4 from few to many	384	406	51.4	18.320^***^
	2 types		175	300	63.2	
	3 types		89	166	65.1	
	≥4 types		45	76	62.8	
**Self-feeling factors**						
Symptoms	Stable	0	404	104	20.5	419.537^***^
	Unstable	1	289	844	74.5	
Side effects	Rarely	0	168	104	38.2	50.997^***^
	Frequently	1	525	844	61.7	
**Family support factors**						
Financial situation	Poverty	0	264	331	55.6	1.751
	Non-poverty	1	429	617	59.0	
Co-resident	No	0	74	96	56.5	0.131
	Yes	1	619	852	57.9	
**Social support factors**						
Medication guidance	Not getting	0	509	715	58.4	0.822
	Getting	1	184	233	55.9	
Medicine location	Specialist hospital	0	479	592	55.3	7.863^***^
	Community	1	214	356	62.5	
Years of participation in community archives	≤ 5	Assign a value of 1–3 from short to long	215	333	60.8	4.337
	6–10		314	424	57.5	
	≥11		164	191	53.8	

** *0.05*,

*** *0.01*.

### Logistic Regression Analysis Results

Table 3 presents the results of logistic regression of the policy's impact on medication dependence with Model 1 and Model 2. The pseudo R-squares of all models are >0.2. Model 1 shows that policy participation has a significant positive impact on medication adherence. The probability that respondents who participate in the policy have good medication adherence is 1.577-times that of those who do not participate in the policy. According to Model 2, the effect of policy participation on medication adherence in the Medication-only group was highly significant, and it was weakly significant in the Subsidy-only group, while it was not significant in the Mixed group. Under the medication-only group model, respondents participating in the policy are 1.735-times more likely to have good medication adherence than non-participants. As well as policy factors, some control variables also have a significant impact on medication adherence. The stability of symptoms showed significant effects in all models; while the family's financial situation, Intellectual Disability with mental disorders, physical condition, and side effects showed significant effects in more models; sex, education, course of disease, medication guidance and medicine location were significant in fewer models.

**Table 3:** Logistic regression model results.

**Variable**	**Model-1: Overall**		**Model-2: service mode**					
			**Medication-only**		**Subsidy-only**		**Mixed**	
	***B***	***OR***	***B***	***OR***	***B***	***OR***	***B***	***OR***
**Policy participation**	0.410^**^	**1.507**	0.606^**^	**1.833**	0.794^*^	**2.212**	0.009	1.009
Sex	0.250^**^	**1.284**	0.316	1.372	0.441	1.554	0.074	1.077
Marital	−0.090	0.914	−0.213	0.808	−0.057	0.945	−0.076	0.926
Education	−0.049	0.952	0.176	1.192	−0.802^***^	**0.448**	0.025	1.025
Professional	0.207	1.230	0.190	1.209	0.712	2.038	0.338	1.402
Diseases(SCH)	−0.012	0.988	−0.465	0.628	0.666	1.947	−0.018	0.983
Diseases(BD)	0.175	1.191	−0.296	0.744	0.831	2.296	0.332	1.394
Diseases(ID-MD)	–0.715^***^	**0.489**	–1.033^**^	**0.356**	−0.136	0.873	−0.620^*^	**0.538**
Physical condition	0.313^*^	**1.367**	−0.137	0.872	−0.126	0.882	1.041^***^	**2.833**
Course of disease	0.056	1.058	0.329^*^	**1.390**	0.251	1.285	−0.176	0.839
Types of medicine	0.071	1.073	−0.004	0.996	0.276	1.318	0.168	1.183
Symptom	–2.473^***^	**0.084**	–2.601^***^	**0.074**	−2.433^***^	**0.088**	−2.771^***^	**0.063**
Side effects	−0.047	0.954	–0.467^*^	**0.627**	−0.412	0.662	0.646^**^	**1.908**
Financial situation	0.335^**^	**1.397**	0.599^***^	**1.821**	0.039	1.040	−0.156	0.855
Co-resident	0.035	1.036	0.342	1.408	0.531	1.701	−0.020	0.981
Medication guidance	−0.216	0.805	–0.403^*^	**0.668**	0.532	1.703	−0.243	0.784
Medicine location	0.151	1.163	−0.189	0.828	−0.275	0.760	0.728^***^	**2.070**
Years of participation in community archives	−0.136	0.873	−0.019	0.981	0.158	1.171	−0.090	0.914
Constant	0.599	1.821	−0.393	0.675	0.016	1.016	0.953	2.594
Pseudo-*R*^2^	0.258		0.267		0.287		0.315	
*N*	1,641		645		316		680	

* *0.1*,

** *0.05*,

*** *0.01*.

*The meaning of the bold values is that the variables are statistically significant*.

## Discussion

Non-adherence to prescribed antipsychotic medication regimens is a significant problem in the treatment of patients with severe mental disorders, especially schizophrenia. It has been estimated that 70–80% of patients with schizophrenia and related psychotic disorders are partially non-adherent (34, 35). This study found that the CFMS as an intervention is effective in improving the medication adherence of community patients. This is consistent with previous studies showing the efficacy of medication adherence interventions (36, 37). Wang et al. shows that because of the CFMS, Beijing has been ranked highest in the regular medication rate of patients with severe mental disorders in 31 provinces in China since 2014 (6). Respondents receiving CFMS have a higher medicine adherence rate of 59.9%, while those who did not receive the service have a medication adherence rate of 48.1%.This is much lower than the result of Gong et al. which was based on the patients of the 686 program in Liuyang, China, and showed that 77% of respondents have good medication adherence (15). There may be two reasons for this. First, the 686 project is a treatment and assistance policy for poor patients, while Beijing's CFMS is a universal policy for all patients with severe mental disorders. Many studies have shown that economy is an important factor affecting the dependence of patients on medication (31, 38). People in poverty-stricken areas, without a fixed source of income, and lack of financial security is more likely to be negatively affected (39). Second, the instrument for measuring medication adherence is different. As above, China's severe mental disorder relief policy began with the 686 project in 2004, which has limited coverage. The policy implications of this finding recommends that local governments introduce a universal policy for all patients with severe mental disorders to take free medicines.

This study also found that the impact of policy on medication adherence of the respondents is not consistent among service modes. It has a significant impact for the respondents of the Medicine-only model group and the Subsidy-only model group, but no significant impact for those of the Mixed mode group. In fact, because of the differences in the design of each model, it also brings different effectiveness for changing health behaviour, which is consistent with previous studies (40, 41). The patients can go to the designated place to receive certain medicines or subsidies every month under the Medicine-only mode and the Subsidy-only mode, while the mixed-mode provides more than two types of services for patients to choose from. For example, in Tongzhou District, patients can take medicines for free in the community, or “see a doctor on their own and be reimbursed in the community at the end of the year,” but the latter has a capped line. There is no capping line in Chaoyang District, but there is a medication list for reimbursement. Volpp et al. demonstrated greater effectiveness in improving adherence to warfarin therapy when financial incentives for patients were structured over a fixed period, rather than for an uncertain time (42). Similarly, when studying patient preferences for medication adherence using financial incentive structures, Hohmann et al. has found that as the receipt of an incentive became more immediate or the probability of the receipt of an incentive became more certain, the effectiveness was greater (43). Obviously, in the current study, compared to the Medicine-only mode and the Subsidy-only mode, the incentive of the Mixed model to patients is not immediate, and the possibility is also uncertain. Two previous meta-analyses on medication adherence intervention have indicated that reinforcement magnitude and frequency should be considered when designing reinforcement interventions to enhance medication adherence. Greater magnitudes of reinforcement engendered larger effect sizes (44, 45).

There are many studies to quantify non-adherence in psychiatric settings and find predictive factors of this phenomenon. The interventions to address these risk factors have also been the focus of much research. This study shows that socio-demographic characteristics, such as sex and education, type of disease, drug side effects, physical condition, and socioeconomic status, are the risk factors of non-adherence. These results are consistent with the previous literature (32, 46). The identification of the above barriers provides the focus of community psychiatrists. This study found that in these severe mental disorders, the Intellectual Disability with mental disorders was significantly associated with medication no-adherence. This disease frequently occurs in adolescence and some patients have taken no medication for many years (32). Therefore, the intervention of CFMS may be ineffective for these patients. It is therefore necessary to increase home visits for such patients and strengthen drug publicity and guidance. In addition, the effects of the control variables in the three modes are different. This shows that the path of policy's influence on patient medication dependence is complicated, and it may also play a moderating role in the influence of other factors. For example, the freedom of choice in the mixed mode allows patients suffering from physical illness or receiving medicines in the community to have more opportunities to contact medical workers and care more about taking medicines. This finding suggests that it is necessary to further explore the path of the free medication policy on medication dependence in the future.

This study makes significant contributions to the current mental health field. Compared with previous studies on the effect of medication adherence intervention based on the clinical intervention experiment, this study is an evaluation of the effects of regional intervention policies. Because 16 districts independently implemented the district's policy trials and formed different policy models, the CFMS policy in Beijing is a natural experiment. Moreover, compared with the literature on the experience of community management of patients with mental disorders in developed countries such as the United States, Germany, and Canada, this article provides an empirical evidence in different research context, which shows that China is a country with low and medium economic development levels, and its political system also has its own characteristics. Last, as far as we know, this study is the first article that specifically evaluated the influence of inclusive CFMS policy (not “686” Program) of China on medication adherence. We not only evaluated the overall impact of Beijing's CFMS policy on medication adherence, but also evaluated the effects of different models in groups.

This study also has some limitations. First, as mentioned above, after the community was selected at random, this study adopted a cluster sampling of all patients in the community. However, the final response rate of the survey was only about 75%. It is possible that survey respondents differ from non-respondents. In particular, most of the non-responders refused home visits by community psychiatrists or had poor social function, and their medication adherence might be worse. Second, this study is a cross-sectional study, which only analyses the current situation of patients with severe mental disorders, and cannot chronologically link the CFMS with patient medication adherence. Third, the measurement of medication adherence in this study used patient reports of adherence in the same way as many other studies. However, Velligan et al. showed that the adherence rate of blood level data (23%) is only less than half of that of participants' self-report data (55%) (47). Therefore, there may be a certain gap between the measurement results of medication dependence in this study and the actual situation.

## Conclusion

In this study, a cross-sectional approach was used to conduct an on-site investigation of representative patients in severe mental communities in Beijing. The impact of the CFMS on patients' medication adherence was objectively evaluated. The facts have proved that this policy, as a policy which benefits people, not only plays a caring role, but also successfully promoted patient adherence behaviours and maintained social stability. Beijing's CFMS model is an improvement and continuation of the 686 project, which has set an example for the whole of China. However, finding an appropriate policy model is a challenge. Exploring intervention measures to solve the risk factors of medication adherence will be the next focus.

## Data Availability Statement

The raw data supporting the conclusions of this article will be made available by the authors, without undue reservation.

## Ethics Statement

The protocol of this study was approved by the Medical Ethics Committee of Capital Medical University (No: Z2020SY123). All respondents were voluntary and written informed consent was obtained. All data collection is anonymous.

## Author Contributions

JZ contributed to the conception and design of the study. JZ, QH, WL, YC, BL, YX, RX, and DL organised the data collection. WL performed the statistical analysis. JZ, QH, and WL wrote sections of the manuscript. All authors contributed to the manuscript revision, read, and approved the submitted version.

## Conflict of Interest

The authors declare that the research was conducted in the absence of any commercial or financial relationships that could be construed as a potential conflict of interest.

## Publisher's Note

All claims expressed in this article are solely those of the authors and do not necessarily represent those of their affiliated organizations, or those of the publisher, the editors and the reviewers. Any product that may be evaluated in this article, or claim that may be made by its manufacturer, is not guaranteed or endorsed by the publisher.
